# Phylogenetic signal in the community structure of host-specific microbiomes of tropical marine sponges

**DOI:** 10.3389/fmicb.2014.00532

**Published:** 2014-10-17

**Authors:** Cole G. Easson, Robert W. Thacker

**Affiliations:** Department of Biology, University of Alabama at BirminghamBirmingham, AL, USA

**Keywords:** coevolution, community ecology, diversity, microbial symbioses, phylogeny, Porifera

## Abstract

Sponges (Porifera) can host diverse and abundant communities of microbial symbionts that make crucial contributions to host metabolism. Although these communities are often host-specific and hypothesized to co-evolve with their hosts, correlations between host phylogeny and microbiome community structure are rarely tested. As part of the Earth Microbiome Project (EMP), we surveyed the microbiomes associated with 20 species of tropical marine sponges collected over a narrow geographic range. We tested whether (1) univariate metrics of microbiome diversity displayed significant phylogenetic signal across the host phylogeny; (2) host identity and host phylogeny were significant factors in multivariate analyses of taxonomic and phylogenetic dissimilarity; and (3) different minimum read thresholds impacted these results. We observed significant differences in univariate metrics of diversity among host species for all read thresholds, with strong phylogenetic signal in the inverse Simpson's index of diversity (*D*). We observed a surprisingly wide range of variability in community dissimilarity within host species (4–73%); this variability was not related to microbial abundance within a host species. Taxonomic and phylogenetic dissimilarity were significantly impacted by host identity and host phylogeny when these factors were considered individually; when tested together, the effect of host phylogeny was reduced, but remained significant. In our dataset, this outcome is largely due to closely related host sponges harboring distinct microbial taxa. Host identity maintained a strong statistical signal at all minimum read thresholds. Although the identity of specific microbial taxa varied substantially among host sponges, closely related hosts tended to harbor microbial communities with similar patterns of relative abundance. We hypothesize that microbiomes with low *D* might be structured by regulation of the microbial community by the host or by the presence of competitively dominant symbionts that are themselves under selection for host specificity.

## Introduction

Marine sponges are globally distributed and perform critical ecological functions in benthic ecosystems (Rützler, 2012; Van Soest et al., 2012). Sponges are active participants in the carbon, nitrogen, and sulfur cycles, performing aerobic and anaerobic processes that benefit the broader community (Taylor et al., 2007; Schläppy et al., 2010; Maldonado et al., 2012; Schöttner et al., 2013). In addition, sponges play a critical role in pelagic–benthic coupling, transferring pelagic carbon and nitrogen to benthic food webs (Lesser, 2006; De Goeij et al., 2013). The diverse communities of microbial symbionts hosted by marine sponges are hypothesized to be the primary drivers of these essential nutrient cycles (Maldonado et al., 2012; Thacker and Freeman, 2012). For example, approximately one-third of Caribbean reef sponges host photosynthetic symbionts (Erwin and Thacker, 2007) that convert dissolved inorganic carbon to organic molecules that are available to heterotrophs, including the sponge host (Freeman and Thacker, 2011). The diversity of sponge-associated microbiomes is unmatched by other invertebrate hosts, such that their complexity is frequently compared to that of mammalian gut microbiomes (Webster et al., 2010; Reveillaud et al., 2014).

Sponges can be broadly classified into two groups based on the abundance of their associated microbial communities. High microbial abundance (HMA) sponges contain diverse and abundant microbial communities that are distinct from the microbial communities found in the surrounding seawater (Hentschel et al., 2003). HMA sponges are also characterized by lower pumping rates and a higher frequency of hosting photosynthetic symbionts (Weisz et al., 2007). Conversely, low microbial abundance (LMA) sponges contain significantly lower abundances of associated microbes that tend to be more similar to the microbial communities found in the surrounding water column (Erwin et al., 2011; Giles et al., 2013). LMA sponges are also characterized by higher pumping rates, with a higher rate of heterotrophic feeding on particulate organic matter (Weisz et al., 2008; Schläppy et al., 2010; Freeman and Thacker, 2011).

Recent work has blurred this distinction between HMA and LMA sponges, emphasizing instead the presence of “core” microbial taxa within symbiotic communities, containing microbiota that are widely shared across diverse sponge hosts, “variable” microbial taxa shared by at least two sponge species, and “host-specific” microbial taxa that are reported from a single sponge species (Schmitt et al., 2011). Studies using a variety of microbial community fingerprinting techniques [such as denaturing gradient gel electrophoresis (DGGE), terminal restriction fragment length polymorphisms (TRFLPs), and automated ribosomal intergenic spacer analysis (ARISA)] as well as clone library sequencing have reported a high degree of host-specificity in both HMA and LMA sponges (Anderson et al., 2010; Erwin et al., 2011, 2012a; Pita et al., 2013; Schöttner et al., 2013; Olson et al., 2014). Quantitative analyses of ARISA data revealed a significant association between microbiome similarity and host sponge species and families (Schöttner et al., 2013), indicating that these communities have likely co-evolved with their hosts. Next generation sequencing (NGS) approaches have increased the precision and quantity of information sampled from sponge-associated microbial communities (Schmitt et al., 2011; Webster and Taylor, 2012; Reveillaud et al., 2014). Multiple studies employing NGS approaches have also demonstrated that sponge microbiomes are largely host-specific, though some seasonal, environmental, and geographic variation has been noted within host species (Hardoim et al., 2012; White et al., 2012; Cleary et al., 2013).

Sponge-specific bacteria, defined as bacterial lineages found only in sponges and not in ambient seawater or sediments, were initially identified through clone library sequencing, but have also been documented using NGS approaches (Taylor et al., 2007, 2012). Together with the direct observation of vertical transmission of some microbial symbionts, these sponge-specific lineages provide additional evidence for co-evolution, and potentially co-speciation, between host sponges and their microbial symbionts (Thacker and Freeman, 2012). However, NGS approaches have also reported “sponge-specific” bacterial lineages from seawater (Taylor et al., 2012). Likewise, more thorough analyses of GenBank sequences have indicated that several bacterial taxa thought to be specific to sponges also occur in other habitats, such as sediment and in other host organisms (Simister et al., 2012; Taylor et al., 2012). While the absolute “sponge-specific” nature of these taxa is debatable, with a recent study suggesting the use of the term “sponge-enriched” instead (Moitinho-Silva et al., 2014), most studies have found the sponge host to be the single strongest influence on the composition of the associated bacterial community (Lee et al., 2010; Webster et al., 2010; Schmitt et al., 2011).

NGS datasets are often extremely large and difficult to manipulate using standard computing power. Limiting the size of the dataset can help remove error and noise, but can also remove meaningful information about rare members of the microbiome (Sogin et al., 2006; Huse et al., 2010). In addition, investigators quantifying the “rare biosphere” have reported evidence of host-specificity even in the extremely rare members of the sponge microbiome (Reveillaud et al., 2014). This pattern holds true even for LMA species, in which a single microbial lineage can dominate host-species-specific microbiomes (Giles et al., 2013).

In the current study, we characterized the diversity and dissimilarity of microbiomes associated with 20 species of tropical marine sponges to test whether host phylogeny significantly impacts symbiotic microbial community structure. We assessed host phylogenetic relatedness using DNA sequences obtained by the Porifera Tree of Life project (Redmond et al., 2013); this approach contrasts with previous comparative studies of sponge microbiomes that relied on taxonomic names to describe host relatedness (Schmitt et al., 2011; Schöttner et al., 2013). We focused our investigation over a relatively narrow geographic range to limit potential biogeographic effects on microbiome community structure. First, we tested whether univariate measures of the diversity of symbiotic microbial communities displayed significant phylogenetic signal across the host phylogeny. Second, we examined both host identity and host phylogenetic relatedness as factors in multivariate analyses of both taxonomic and phylogenetic dissimilarity among microbiomes to determine whether host relatedness influences microbiome community structure in addition to host identity. Finally, we investigated how measures of diversity and dissimilarity change when using different read count thresholds.

## Materials and methods

### Sample collection and DNA extraction

We collected tissues from 100 sponge specimens representing 20 host species (5 specimens per species) by snorkeling and using SCUBA at several shallow dive sites near Bocas del Toro, Panama, between 2006 and 2012 (Supplementary Table 1). Species identities were confirmed by microscopic examination of morphological characters (Hooper and van Soest, 2002). Samples were collected into sterile bags, then preserved in 95% ethanol at the Smithsonian Tropical Research Institute (STRI) and stored at 4°C until extraction. DNA was extracted from combined ectosomal and choanosomal tissue using the PowerSoil DNA isolation kit (MoBio Laboratories, Inc.), following the standard EMP protocol (http://www.earthmicrobiome.org/emp-standard-protocols/dna-extraction-protocol/).

### Next-generation sequencing

Sequencing of the samples in our study was completed in collaboration with other researchers as part of the EMP (http://www.earthmicrobiome.org/). Our collaborators at EMP amplified and sequenced the V4 region of the 16s rRNA gene using the bacterial/archaeal primer pair 515F/860R and following previously published methods (Caporaso et al., 2012). Amplicons were fused to Illumina barcodes and sequencing was completed on an Illumina platform.

### Quality control, filtering, and taxonomic assignments

Raw sequences were quality-filtered (average quality score = 30, window size = 5 bases, maximum number of homopolymers = 8) and trimmed to a minimum length of 100 base pairs. We removed 10 samples from our dataset that did not meet these quality standards. We aligned the sequences to a trimmed SILVA database (v102, trimmed to the V4 region 11894–25319; Schloss et al., 2009). The aligned sequences were then checked for chimeras, removing all that were found. Sample sequences were then classified based on the SILVA reference database, with a minimum cutoff of 60% identity. The classified sequences were clustered into operational taxonomic units (OTUs) using a 97% similarity cutoff, yielding a data table containing each sample and its respective OTUs.

We extracted the 90 samples specific to our study from the full EMP dataset using four custom Perl scripts (Supplementary File 1). We used the first script (matchRows.pl) to extract specific rows (those containing the pertinent samples) from the full EMP dataset based on user-provided criteria. We used the second script (RemoveColumnByThreshold.pl) to remove all columns with a column sum of zero from the extracted rows (i.e., deleting OTUs that were not found in samples specific to the current study). Since the second script allowed users to set any value for column sums, we also used this script to reduce the dataset to specific sequence read thresholds. We used the third script (SavedOTUdatabase.pl) to match the new OTU occurrence matrix with the OTU database file obtained from mothur, generating a reduced OTU database file, which contained the OTU identifier, the OTU sequence, and the taxonomic classification of each OTU. Finally, we used a fourth script (deleteSpecificColumns.pl) to remove metadata columns not needed for analyses in *R*.

We performed statistical analyses on three versions of the same dataset, using minimum OTU read thresholds (i.e., the minimum number of reads required for a particular OTU to be included in the dataset) of 1, 5 (Supplementary File 2), and 500 reads. To reduce the size of the dataset, and remove noise and potential error, we focus the remainder of the text on the analysis of the dataset with a minimum read threshold of 500 reads. The use of minimum read threshold values is considered a more conservative approach to standardizing an NGS dataset compared to approaches such as regularization and convex minimization (Dunn et al., 2013).

### Microbial community diversity

Using the R package vegan (Oksanen et al., 2014), we converted OTU abundance to relative abundance to minimize the possibility of false positives in our analyses (McMurdie and Holmes, 2014). We also used vegan to calculate three univariate measures of the diversity of the microbial community associated with each host specimen: OTU richness (*S*), the Shannon–Weaver index (*H*′), and the inverse Simpson's index (*D*). We compared these metrics among host species using analyses of variance (ANOVA).

### Phylogenetic reconstructions

We constructed a phylogeny of sponge hosts by obtaining sequences of the gene encoding the small subunit (18S) of nuclear ribosomal RNA for each host species from GenBank (Supplementary Table 2). We aligned the sequences using the default options of MAFFT 7.017 (Katoh et al., 2002), as implemented in Geneious 6.1.6 (Biomatters Limited). We constructed the host phylogeny by implementing a relaxed-clock model in MrBayes version 3.2.1 (Ronquist et al., 2012), employing the computational resources of iPLANT (Goff et al., 2011). The options set in MrBayes included constraining the clade containing the genera *Aiolochroia, Aplysina*, and *Chondrilla* (all members of subclass Myxospongiae) as an outgroup and implementing the independent gamma rate relaxed clock model with a birth–death process. This analysis included three parallel runs of 10 million generations, each using four Markov chains and sampling every 100 generations. We assessed convergence of the chains by examining the average standard deviation of split frequencies, which reached a value of 0.003. Following a burn-in of 25%, we summarized the output of the three runs as a consensus phylogeny.

To enable analyses of microbiome phylogenetic dissimilarity, we constructed a maximum likelihood phylogeny of bacterial OTUs. We aligned OTU sequences using the default options of MAFFT and constructed the phylogeny using Fasttree2 (Price et al., 2010), as implemented by iPLANT, using the default settings.

### Phylogenetic signal

Phylogenetic signal describes the degree to which more closely related organisms share more similar traits (Blomberg et al., 2003). We used the phylosignal function of the R package picante (Kembel et al., 2010) to test whether *D* displayed significant phylogenetic signal given the host sponge phylogeny (i.e., whether more similar values were associated with more closely related hosts more often than expected by chance).

### Taxonomic and phylogenetic dissimilarity

We calculated microbial community taxonomic dissimilarity among specimens using the Bray–Curtis index of dissimilarity (BCD). We calculated mean BCD among specimens within host species to assess the variability of microbiomes within host species. We compared these values between LMA and HMA sponges using a *t*-test, designating LMA/HMA status based on previous studies (Weisz et al., 2007).

We used the R package picante (Kembel et al., 2010) to calculate phylogenetic dissimilarity among microbiomes, which reflects the genetic variation among the microbial OTUs present in each community. This analysis was only conducted on the two reduced datasets, as the original dataset yielded a phylogenetic distance matrix that exceeded the integer limit of R. We used the adonis function of the R package vegan (Oksanen et al., 2014) to quantify the impact of host species identity on BCD and phylogenetic dissimilarity. Since adonis could not simultaneously treat host identity and host phylogeny as factors, we used Mantel tests to assess the correlation between each of these individual factors and BCD, as well as a partial Mantel test to assess the effect of host phylogeny on BCD given host identity. We conducted similar Mantel tests to examine the correlations between host identity, host phylogeny, and microbial phylogenetic dissimilarity.

We calculated the percentage contribution to BCD of specific OTUs using SIMPER (Oksanen et al., 2014) for only the 390 OTUs present given a minimum threshold of 500 reads. Since SIMPER can only perform pairwise comparisons, the microbial community of each host species was compared to the microbial community of the remaining hosts pooled together, thereby contrasting an individual host species to all other hosts species and placing emphasis on the OTUs unique to each host. The output of this analysis revealed the percentage contribution of each OTU to this contrast. When employing lower minimum read thresholds, individual microbial OTUs excluded by the 500 read threshold contributed nearly zero percent to host species contrasts.

### Effect of read thresholds

After filtering the dataset by using minimum read threshold values of 1, 5, 10, 50, 100, 500, 1000, and 5000, we used ANOVA to calculate the *F*-ratio associated with variation in *S* among host species. We used a polynomial regression to test whether these *F*-ratios were significantly related to threshold values.

### Reproducibility of analyses

All statistical analyses were performed in R v. 3.1.1. Supplementary File 3 contains a set of R commands that allow the user to reproduce all of the analyses described in this manuscript.

## Results

**Figure 1:**
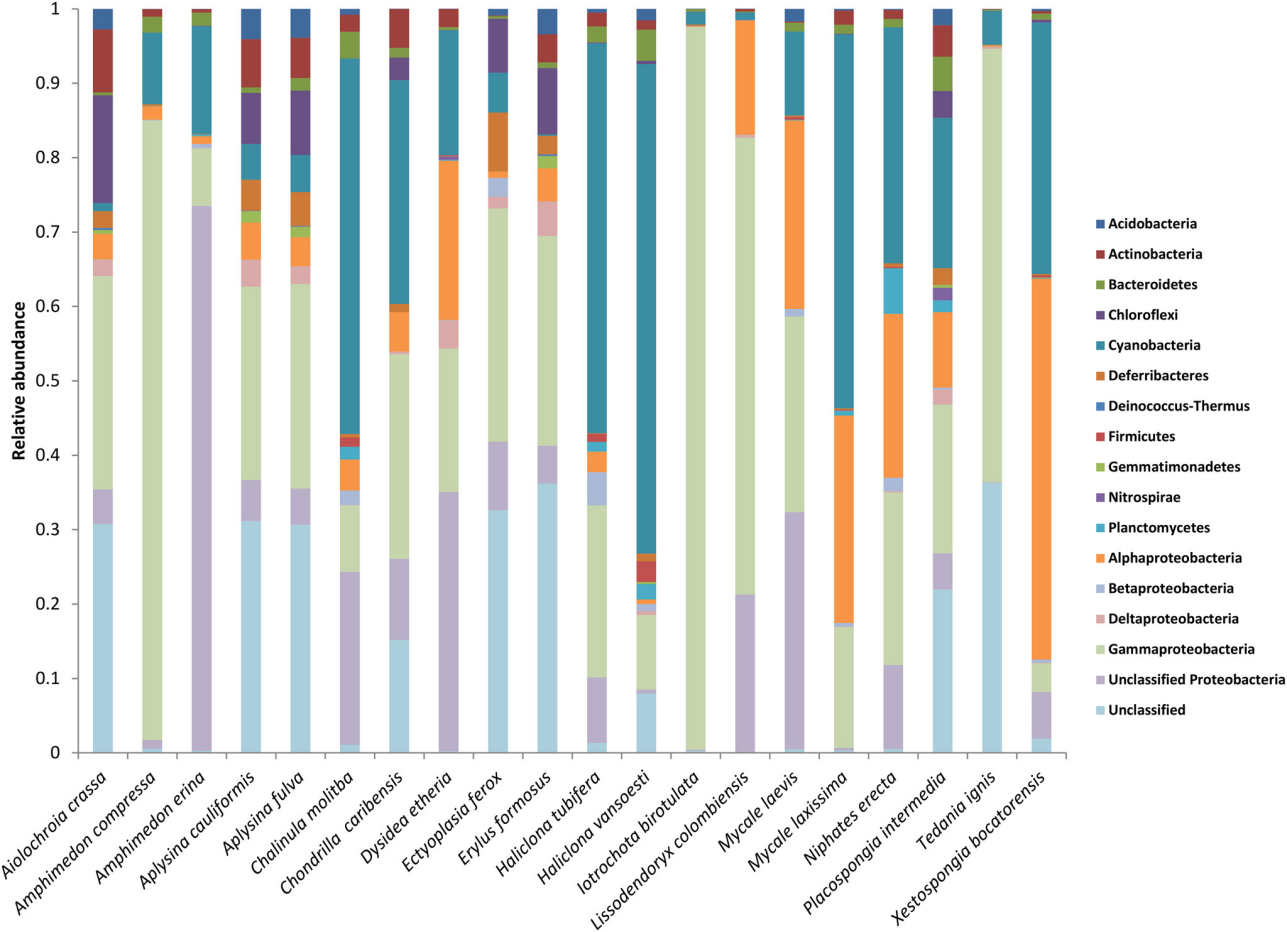
**Mean relative abundance of microbial taxa within each host species**. Microbial phyla are displayed to the right of the chart, with the phylum Proteobacteria split into classes.

The raw data for this EMP study are available at http://www.earthmicrobiome.org/. From the starting set of 100 sponge specimens, 90 specimens met all quality control standards, yielding 88,395 unique OTUs (defined as 97% sequence similarity) representing 20 bacterial phyla (based on SILVA classification), with a maximum of 8357 unique OTUs in a single host specimen. Minimum thresholds of 5 and 500 reads per OTU yielded 21,395 and 390 unique OTUs, respectively. Proteobacteria was the most abundant microbial phylum, accounting for approximately 47 % of all unique OTUs, consistent with previous studies investigating sponge microbial communities (Figure 1). Other notably abundant phyla included Actinobacteria, Chloroflexi, and Cyanobacteria. A few host species displayed surprisingly low phylum-level diversity, including *Iotrochota birotulata*, *Tedania ignis*, and *Lissodendoryx colombiensis*, while others hosted high phylum-level diversity, including the verongid species *Aiolochroia crassa*, *Aplysina cauliformis*, and *Aplysina fulva*. Classification of these microbial communities according to the criteria of Schmitt et al. (2011) revealed that only 1.5% of the community consisted of “core” taxa and only 11% could be considered “host-specific” taxa. The majority of the microbial community in our sample set occurred in several host species, but not ubiquitously. Interestingly, ten of the twenty host species contained no species-specific microbial OTUs, including four of the seven HMA species. In addition, within some host species, a relatively large percentage of OTUs were not classified when referencing the SILVA database. For example, at a minimum threshold of 500 reads, 91 of 390 OTUs (23%) were not classified. After referencing the Greengenes database (DeSantis et al., 2006), 10 of these 91 reads could be classified as Archaea, 67 as Bacteria, and 14 remained unclassified.

**Figure 2:**
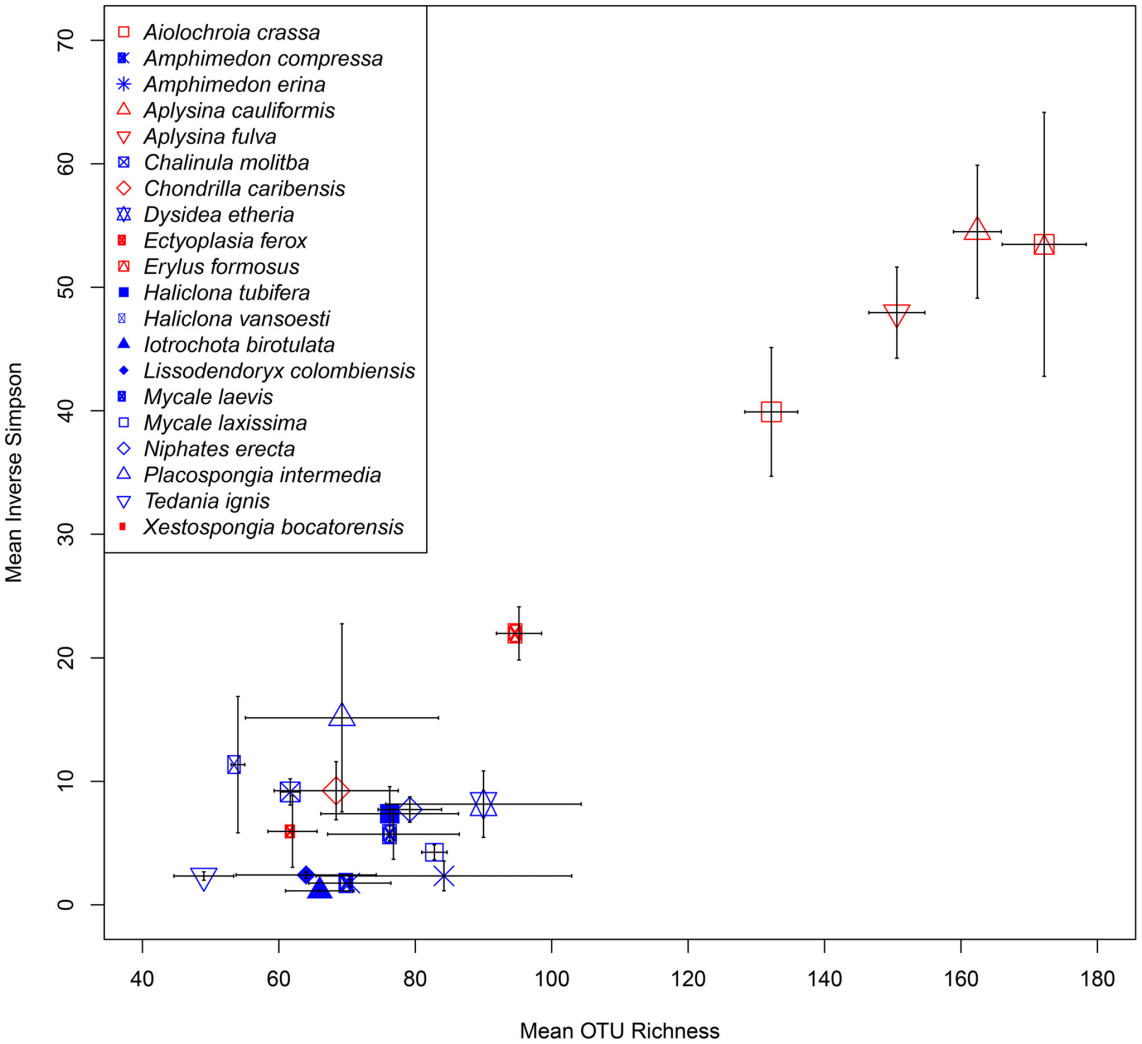
**Scatterplot of mean (± SE) OTU richness (*S*) and inverse Simpson's index (*D*) for each sponge host**. High microbial abundance (HMA) and low microbial abundance (LMA) classifications are displayed as red and blue symbol colors, respectively.

At a minimum threshold of 500 reads, the mean OTU richness (*S*) of sponge microbiomes ranged from 811 in *T. ignis* to 5263 in *Erylus formosus* (Table 1; summaries for minimum thresholds of 1 and 5 reads are presented in Supplementary Tables 3, 4, respectively). Comparisons among host species revealed significant differences in *S*, (ANOVA: *df* = 19, *F* = 17.82, *P* < 0.001), *H*′ (ANOVA: *df* = 19, *F* = 24.46, *P* < 0.001), and *D* (ANOVA: *df* = 19, *F* = 14.31, *P* < 0.0001). We observed significant differences in these univariate metrics for all read thresholds (Supplementary File 2). A plot of mean OTU richness vs. the inverse Simpson index (Figure 2) provided a visualization of the substantial variation in these metrics among host species. Four HMA host species with high values of these metrics were clearly separated from a cluster of LMA host species with low values; however, two HMA host species (*Chondrilla caribensis* and *Xestospongia bocatorensis*) were similar to the LMA host species. Notably, both of these host species contain abundant populations of photosynthetic bacteria (Erwin and Thacker, 2007).

**Table 1:** **Mean ± standard error of univariate measures of microbiome diversity for each host species, analyzed with a minimum threshold of 500 reads, and defining OTUs by 97% sequence similarity**.

**Species**	***S***	***H*′**	***D***	**Within-host BCD**	**SIMPER OTUs**	***n***
*Aiolochroia crassa*	132.2 ± 3.88	4.09 ± 0.07	39.91 ± 5.21	33.7 ± 7.9	25	5
*Amphimedon compressa*	70.4 ± 6.02	1.17 ± 0.28	1.76 ± 0.28	16.2 ± 5.1	1	5
*Amphimedon erina*	84.2 ± 18.74	0.91 ± 0.51	2.34 ± 1.2	33.7 ± 19.2	2	5
*Aplysina cauliformis*	162.4 ± 3.50	4.39 ± 0.05	54.5 ± 5.38	26 ± 6	27	5
*Aplysina fulva*	150.6 ± 4.11	4.24 ± 0.04	47.95 ± 3.69	27.7 ± 6.6	25	5
*Chalinula molitba*	61.67 ± 1.45	2.77 ± 0.12	9.14 ± 1.06	24.1 ± 10.8	9	3
*Chondrilla caribensis*	68.4 ± 9.10	2.71 ± 0.15	9.24 ± 2.35	29.2 ± 9.1	12	5
*Dysidea etheria*	90 ± 14.32	2.49 ± 0.30	8.16 ± 2.69	66.4 ± 15.8	15	5
*Ectyoplasia ferox*	95.2 ± 3.31	3.44 ± 0.07	21.98 ± 2.15	24.6 ± 6.2	17	5
*Erylus formosus*	172.2 ± 6.16	4.38 ± 0.12	53.48 ± 10.69	29.7 ± 7	30	5
*Haliclona tubifera*	76.25 ± 10.09	2.38 ± 0.46	7.38 ± 2.19	52.5 ± 18.3	9	4
*Haliclona vansoesti*	54 ± 1.00	2.95 ± 0.26	11.35 ± 5.52	23.9 ± 19.5	11	2
*Iotrochota birotulata*	66 ± 5.02	0.4 ± 0.11	1.13 ± 0.04	4 ± 1.5	1	4
*Lissodendoryx colombiensis*	64 ± 10.28	1.2 ± 0.08	2.42 ± 0.25	42.4 ± 18.5	4	5
*Mycale laevis*	76.8 ± 9.65	2.06 ± 0.26	5.72 ± 2.03	48.8 ± 15.6	6	5
*Mycale laxissima*	82.8 ± 1.85	2.11 ± 0.18	4.26 ± 0.61	51.8 ± 13.2	8	5
*Niphates erecta*	79.2 ± 4.65	2.61 ± 0.12	7.72 ± 1.03	41.4 ± 10.4	10	5
*Placospongia intermedia*	69.25 ± 14.05	2.75 ± 0.47	15.15 ± 7.62	73 ± 21.8	17	4
*Tedania ignis*	49 ± 4.38	1.08 ± 0.11	2.33 ± 0.34	35.5 ± 9.9	3	5
*Xestospongia bocatorensis*	62 ± 3.61	2.02 ± 0.50	5.95 ± 2.90	35.9 ± 17.8	6	3

S, OTU richness; H′, Shannon index; D, inverse Simpson index; within-host BCD, intraspecific percentage Bray–Curtis dissimilarity; SIMPER OTUs, number of OTUs explaining 40% of Bray–Curtis dissimilarity; n, sample size.

**Figure 3:**
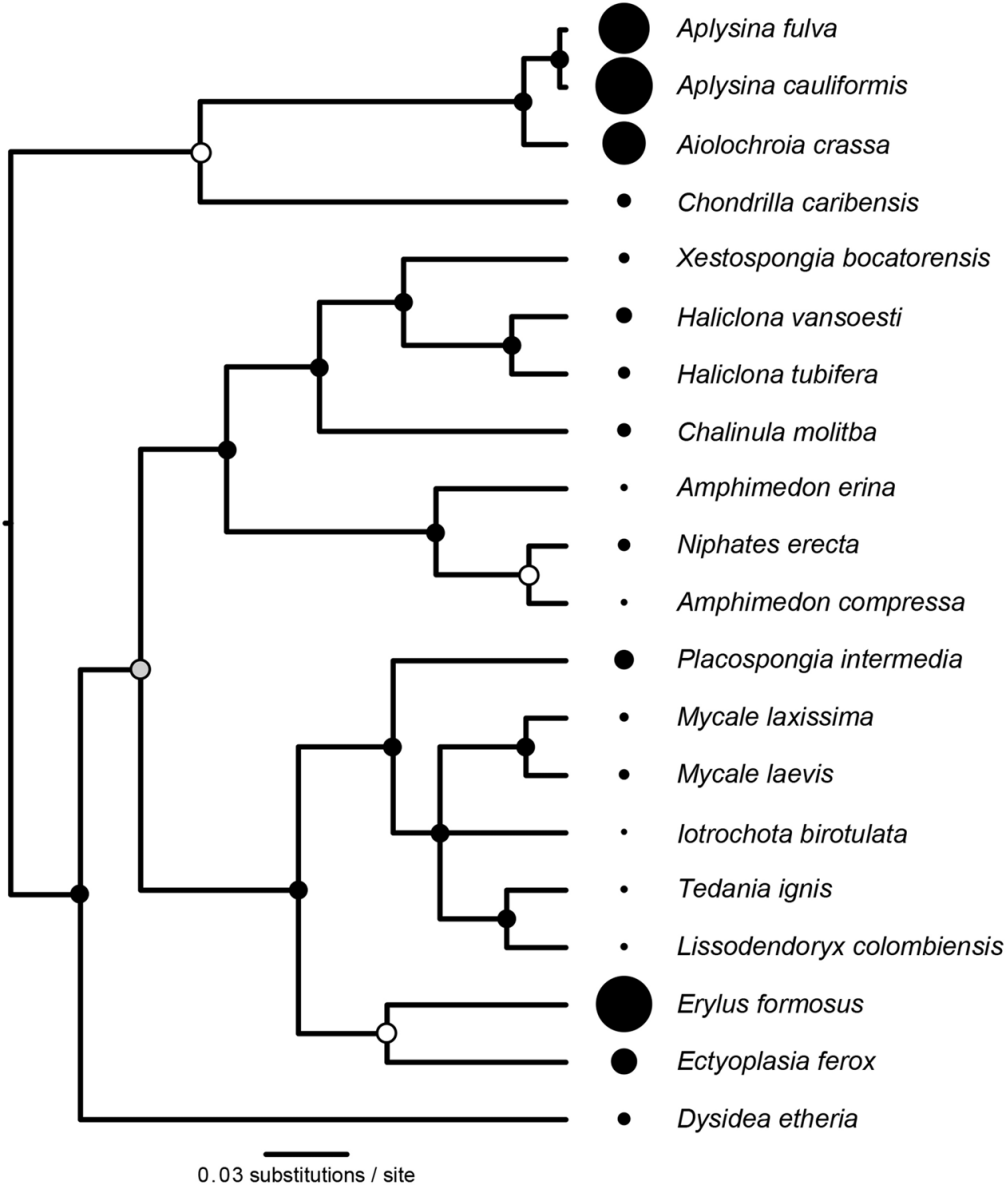
**Phylogenetic signal in the inverse Simpson's index (*D*)**. The phylogeny of host sponge species is based on 18S rRNA gene sequences. The scale bar indicates the number of nucleotide substitutions per site, while circles at the nodes of the phylogeny indicate percentage Bayesian posterior probabilities (PP): black, 100% PP; gray, 95–99% PP; white, <95% PP. Circles at the tips of the phylogeny are sized in proportion to the average value of the inverse Simpson's index (*D*) for each host (Table 1), which displayed significant phylogenetic signal (*K* = 0.591, *P* = 0.003).

The reconstructed phylogeny of host species (Figure 3) was a well-supported subset of the phylogeny presented by Redmond et al. (2013). We found significant phylogenetic signal in *D* (*K* = 0.591, *P* = 0.003, Figure 3), with three representatives of order Verongida (*A. cauliformis*, *A. crassa*, and *A. fulva*), along with *E. formosus* (order Astrophorida), all displaying relatively high values of *D*, while five representatives of order Poecilosclerida (*I. birotulata*, *L. colombiensis*, *Mycale* spp., and *T. ignis*) all displayed very low values of *D*.

**Figure 4:**
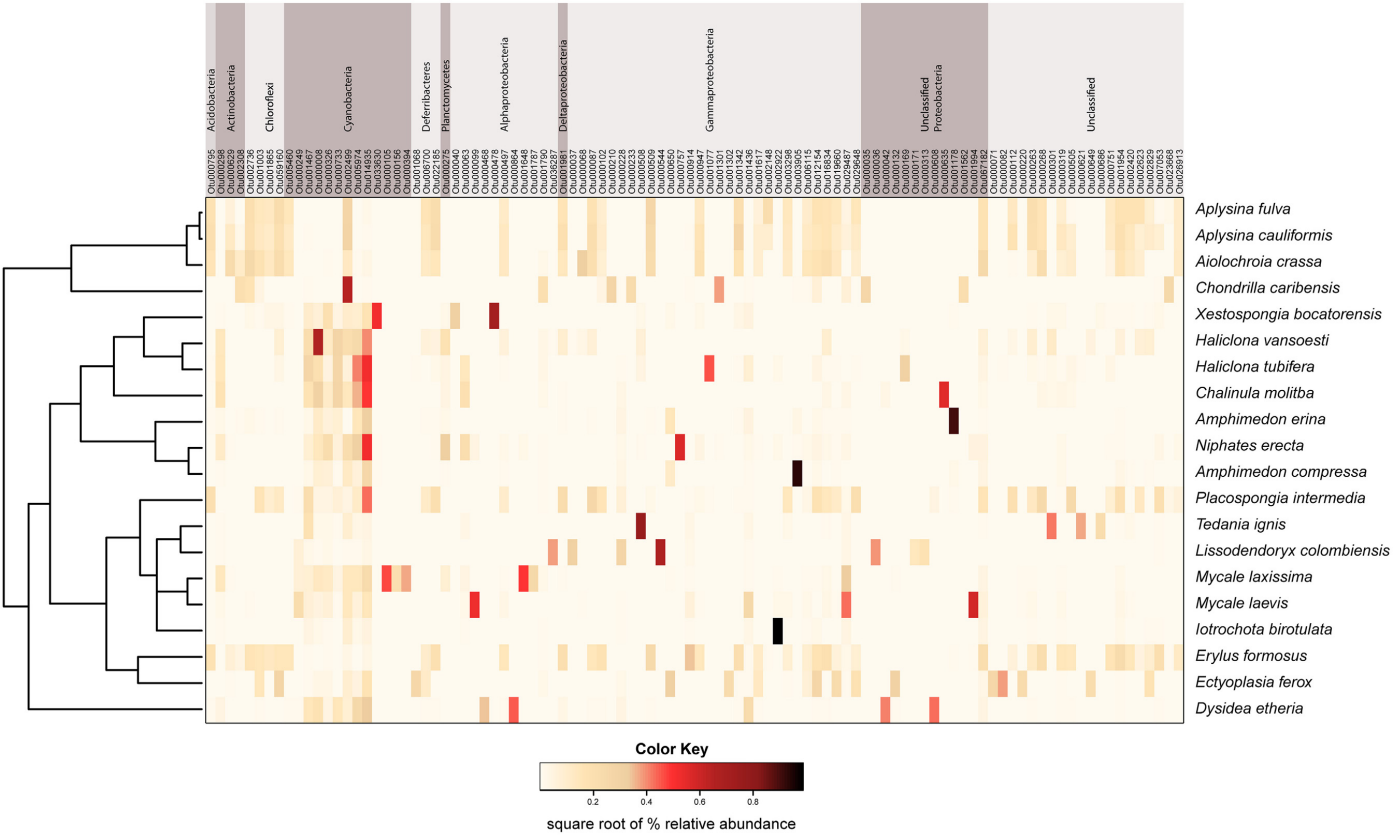
**Mean relative abundance heat map of the 100 most abundant microbial OTUs in each host sponge species**. These data are square-root transformed for ease of visualization. OTUs are grouped by phylum across the top of the figure, with the exception of Proteobacteria, which is split into classes. The host sponge phylogeny is displayed to the left of the heat map for ease of reference.

**Figure 5:**
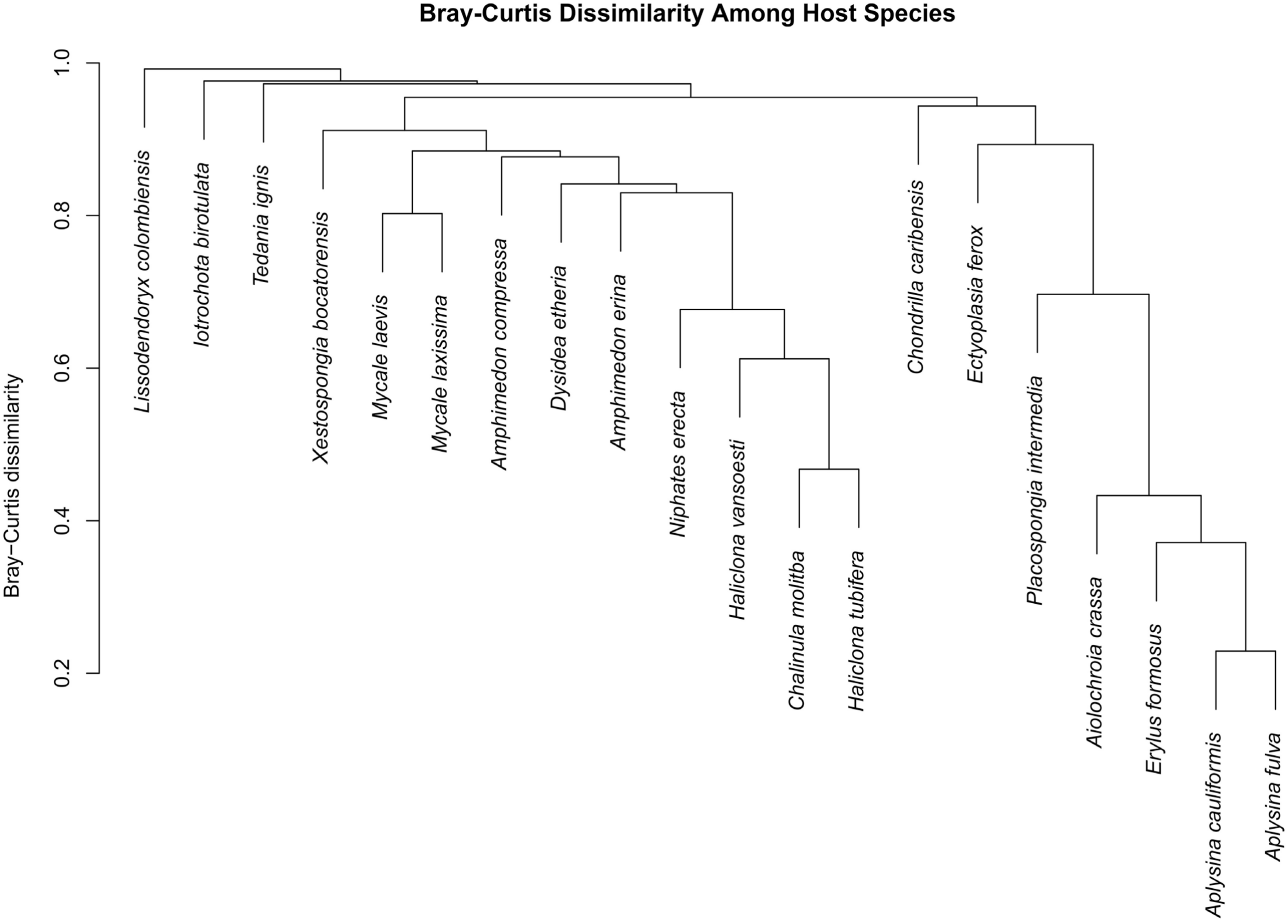
**Dendrogram displaying Bray–Curtis dissimilarity among host sponge species**. Host species exhibited varying degrees of dissimilarity from one another, but all hosts were significantly different from each other (adonis: *df* = 19, *F* = 10.241, *R*^2^ = 0.735, *P* < 0.001).

We observed a wide range (4–73%) of within-host-species variability in BCD (Table 1); surprisingly, this variability was not related to the HMA or LMA classification of the host species (mean ± SE, HMA: 29.5 ± 1.5, LMA: 39.5 ± 5.4; *t* = 1.768, *df* = 14, *P* = 0.099). LMA or HMA classification also had no effect on the number of unique OTUs found in a particular host species (mean ± SE, HMA: 2.4 ± 1.3, LMA: 1.9 ± 0.6; *t* = 0.35, *df* = 9, *P* = 0.737). We visualized variation in community structure among host species using both a heat map of the relative abundance of the 100 most abundant OTUs (Figure 4) and a hierarchical clustering dendrogram displaying average linkages among host species (Figure 5). Analysis using adonis provided strong support for the effect of host identity on BCD (adonis: *df* = 19, *F* = 10.241, *R*^2^ = 0.735, *P* < 0.001). We also used adonis to perform a *post-hoc* comparison of three verongid hosts (*A. cauliformis*, *A. crassa*, and *A. fulva*) that contained visually similar communities (Figure 4). Despite the high phylogenetic relatedness of these hosts, and their similar values of *D* (Figure 3), the microbiomes of these three host species displayed highly significant differences in BCD (adonis: *df* = 2, *F* = 4.62, *R*^2^ = 0.435, *P* < 0.001). Analysis using Mantel tests found that, when tested individually, host identity (Mantel: *r* = 0.422, *R*^2^ = 0.178, *P* < 0.001) and host phylogeny (Mantel: *r* = 0.602, *R*^2^ = 0.362, *P* < 0.001) each explained a significant amount of variability in BCD. Testing the effect of host phylogeny given host identity greatly reduced the explanatory power of phylogenetic relatedness, but remained significant (Partial Mantel: *r* = 0.182, *R*^2^ = 0.033, *P* < 0.001).

The phylogeny of microbial OTUs (Supplementary File 4) constructed for analyses of phylogenetic dissimilarity is not the true phylogeny of these microbial taxa, but instead represents the genetic variation present in the microbial communities and is appropriate for analyses of beta-diversity (Hamady and Knight, 2009). Microbial phylogenetic dissimilarity was significantly impacted by host identity (adonis: *df* = 19, *F* = 57.541, *R*^2^ = 0.940, *P* < 0.001, Table 2). Analysis using Mantel tests revealed that when tested individually, host identity (Mantel: *r* = 0.331, *R*^2^ = 0.109, *P* < 0.001) and host phylogeny (Mantel: *r* = 0.382, *R*^2^ = 0.146, *P* < 0.001) each explained a significant amount of phylogenetic dissimilarity. Testing the effect of host phylogeny given host identity reduced the explanatory power of phylogenetic relatedness, but remained significant (Partial Mantel: *r* = 0.268, *R*^2^ = 0.072, *P* < 0.001). BCD and phylogenetic dissimilarity are not necessarily independent of one another, and these two metrics were significantly correlated (Mantel test, *r* = 0.660, *R*^2^ = 0.436, *P* < 0.001). SIMPER analyses revealed the OTUs specific to each host species that were the primary drivers of the observed differences in BCD and phylogenetic dissimilarity (Supplementary Figure 1). The number of bacterial taxa comprising 40% of the observed BCD variation ranged from 1 to 30 OTUs among host species and reflected observed trends in *D* (Table 1).

**Table 2:** **Analyses of Bray–Curtis dissimilarity and phylogenetic dissimilarity among host species using the *R* function adonis**.

	***df***	**Sum of squares**	**Mean squares**	***F*-ratio**	***R*^2^**	***P*-value**
**BRAY–CURTIS DISSIMILARITY**						
Host species identity	19	28.47	1.498	10.241	0.735	<0.001
Residuals	70	10.242	0.146		0.265	
**PHYLOGENETIC DISSIMILARITY**						
Host species identity	19	1.72162	0.091	57.541	0.940	<0.001
Residuals	70	0.166	0.0023		0.088	

We observed significant differences in *S* among host species across an array of minimum read thresholds, and the *F*-ratio of this test was significantly impacted by minimum read threshold (polynomial regression: *df* = 2, *F* = 24.03, *P* = 0.006; Supplementary Figure 2). In all cases, *S* displayed a significant amount of variability among hosts, indicating that the effect of host identity on *S* is robust. The significance of the *F*-ratio varied among minimum read thresholds across all three diversity indices, but was most substantial when comparing *S*. The effect of host species identity was highest for all diversity metrics at a minimum threshold equal to or greater than 500 reads (Supplementary File 2).

## Discussion

Previous researchers have used a wide variety of techniques to document that sponge-associated microbial communities are largely host specific (e.g., Erwin et al., 2012a; Reveillaud et al., 2014), but host phylogenetic relatedness has only rarely been included as a specific factor influencing microbiome community structure (Schöttner et al., 2013). Our analysis of the microbiomes of 20 host taxa over a narrow geographic range adds further evidence to the high degree of host specificity observed in these microbial communities. Host identity and host phylogeny were each significant individual influences on Bray–Curtis dissimilarity (BCD) and phylogenetic dissimilarity; however, when examined together, host identity explained much more variance than host phylogeny. In our dataset, this outcome is largely due to closely related host taxa harboring extremely different microbiomes. Despite these striking differences in microbial community composition, one aspect of community structure, the inverse Simpson index of diversity (*D*), displayed significant phylogenetic signal across the host phylogeny. *D* is frequently described as an index of dominance because it is most strongly influenced by the relative abundance of the most common taxa in a community (Magurran and Magurran, 1988; Haegeman et al., 2014). Thus, although the identity of specific microbial OTUs varied substantially among host sponges, more closely related sponge species tended to harbor microbial communities with more similar patterns of relative abundance and dominance.

Early studies of sponge-microbe associations investigated fewer host species and used methods such as clone library construction that identified fewer OTUs per host; however, several of these early studies proposed the hypothesis of a uniform microbial community associated with sponges (Hentschel et al., 2002, 2006; Hill, 2004; Montalvo and Hill, 2011). Later studies proposed the occurrence of sponge-specific “sequence clusters” in phylogenies of microbial taxa (Taylor et al., 2007; Thiel et al., 2007; Webster et al., 2010; Simister et al., 2012), indicating that, in many cases, sponge-associated bacteria were found in monophyletic groups. Continued work on this topic has provided strong support for the hypothesis that microbial communities are largely host-species-specific (Taylor et al., 2004; Erwin et al., 2012b; Pita et al., 2013), while placing less emphasis on the occurrence of sponge-specific lineages and instead describing these taxa as “sponge-enriched” (Taylor et al., 2012; Moitinho-Silva et al., 2014). The host-specific nature of sponge-associated microbial communities is now well-established, and next-generation sequencing techniques continue to document this specificity in an increasing number of host taxa (Lee et al., 2010; Schmitt et al., 2011; Reveillaud et al., 2014).

In a strict sense, the terms HMA and LMA refer to the abundance of microbes resident within a sponge host, but these terms are often used to infer characteristics of diversity and microbial specificity (Weisz et al., 2007), with HMA sponges being associated with highly diverse communities (Schmitt et al., 2008; Erwin et al., 2012a) and highly specific communities (Hentschel et al., 2003; Schläppy et al., 2010; Gerçe et al., 2011). Furthermore, several LMA sponges have previously been hypothesized to be more reflective of the surrounding environment than HMA sponges (Weisz et al., 2007; Erwin et al., 2011). In our study, we were surprised to observe strong host specificity even in sponges characterized as LMA species. Giles et al. (2013) also found a large amount of specificity in LMA hosts. Our investigation demonstrated that several LMA sponge species harbor communities with moderately high OTU richness, while some HMA species host microbiomes with considerably lower OTU richness. The two HMA species hosting the lowest OTU richness, *C. caribensis* and *X. bocatorensis*, both host dense populations of photosynthetic cyanobacteria (*Synechococcus spongiarum* and *Oscillatoria spongeliae*, respectively; Thacker and Freeman, 2012). However, it is unclear whether these photosymbionts can structure the remainder of the microbiome, since two other HMA species hosting *S. spongiarum* (*A. cauliformis* and *A. fulva*) displayed among the highest values of OTU richness. In addition, some LMA hosts displayed extremely low values of *D*, indicating that these sponges were not hosting a random microbial assemblage; instead, there seems to be strong evolutionary selection for some sponge lineages to host an extremely specific microbial community that is dominated by a relatively low number of OTUs. These results are similar to those of Poppell et al. (2013), who used DGGE banding patterns to assess diversity in a set of 8 HMA and 7 LMA species and observed significantly lower diversity (and values of *D*) in the LMA species.

We employed multivariate approaches to further explore the nature of these microbial associations. High levels of community dissimilarity are often noted between host sponges (Lee et al., 2010; Reveillaud et al., 2014), and although not often directly tested, dissimilarity often decreases within taxonomic and phylogenetic groupings. This observation is also suggestive of a phylogenetic signal in the structuring of microbiomes. Schöttner et al. (2013) tested this idea directly and noted a significant effect of host species and family on the types of microbial taxa found in specimens of species within the family Geodiidae. When testing the influence of phylogenetic or taxonomic relatedness, it is most appropriate to either test taxonomic groups as nested factors or to use a phylogenetic or taxonomic distance matrix as a factor (Kembel et al., 2010). We used adonis to assess the impact of host identity on microbiome community and phylogenetic dissimilarity, finding that this factor accounted for the majority of variation in these measures. However, adonis could not simultaneously estimate the impact of host identity and host phylogeny (or host relatedness). To assess the relative impact of these factors, we used a partial Mantel test, finding that host phylogeny explained very little variation in community dissimilarity after accounting for host identity. These data suggest that the strong selective forces for divergent microbiome community composition remain strong even among closely related hosts, suggesting that symbiotic microbes might play critical roles in niche differentiation among host species.

We observed an extremely wide range of intraspecific variability in community structure, with some LMA species displaying less than 5% BCD and others displaying more than 50% BCD among individuals. Surprisingly, this range was not correlated with the HMA or LMA classification of the host species. Thus, although some LMA species with extremely high intraspecific variability might be more reflective of the surrounding environment, other LMA species appear to be under strong selective pressures to limit membership in their microbiomes. Furthermore, our sampling strategy focused on representing both ectosomal and choanosomal tissue from each specimen. Species with high intraspecific variability, such as *Dysidea etheria* and *Placospongia intermedia* (Table 1), might reflect zonation of microbial symbionts among microhabitats within the host. Future studies could explicitly test this hypothesis of microbiome zonation by carefully excising distinct tissue layers and cell types.

SIMPER analysis of BCD highlighted the wide variation in host-microbial associations. Host species with high values of *D* harbored more even communities, where no one OTU accounted for a large proportion of the BCD (Supplementary Figure 1). Conversely, some host species were dominated by one or a few microbial taxa, and these specific OTUs contributed to a large proportion of the contrast of BCD among species (Supplementary Figure 1). Indeed, the 5 highest proportional contributions of single OTUs were observed in 5 LMA species (*A. compressa, A. erina, I. birotulata, L. colombiensis*, and *T. ignis*). Importantly, these proportional contributions are not necessarily related to unique membership in a particular community. Though all of the sponge species in our study hosted significantly dissimilar communities, half of these species possessed no “species-specific” microbial taxa. Given the strong statistical signal for host identity, our results suggest that the observed significant dissimilarity among host species was largely driven by differences in relative abundance, with each host species harboring specific microbial assemblages rather than strictly unique OTUs. This pattern was also reported by an earlier study by Erwin et al. (2012a), which described this type of community structure as a “specific mix of generalists.”

We found that host identity maintained a strong statistical signal at all minimum read thresholds tested in our study. The significance of host identity decreased with lower minimum read thresholds, revealing that the microbial OTUs that distinguish hosts from one another, although not necessarily unique to a particular host, are often among the most dominant members of their community. This finding suggests that removing OTUs with lower abundance reduced noise in our dataset, likely due to the presence of microbes found more broadly in the community. Additionally, increasing the minimum read threshold added confidence to our analysis by ensuring that the observed taxa are of biological origin, and not a product of error (Reveillaud et al., 2014). The relevance of rare microbial OTUs in large NGS datasets is still an area of much debate and the use of minimum read thresholds is considered a conservative way to reduce false positives while maintaining the majority of the biological diversity (Dunn et al., 2013).

In addition to the statistical advantages of using minimum read thresholds, some practical issues must be considered when analyzing NGS datasets, since these data are often extremely large and are potentially unmanageable without significant computing power. In our dataset, limiting the minimum read threshold to 5 reads reduced the number of OTUs by 76%. This reduction not only reduced the amount of computing power needed to process these data, but it also permitted us to conduct community phylogenetic analyses in *R*. Although the full dataset generated phylogenetic distance matrices that far exceeded *R*'s current integer limit (R Development Core Team, 2008), analyses of the reduced dataset still exceeds most standard computing power. Our study made use of the cyber-infrastructure provided by iPlant to perform analyses on a super-computing platform. As Internet-based tools such as iPlant become more widely available, these limitations will become less important, but the practical processing of these large datasets remains a challenge today.

Our results lead us to consider the designations LMA and HMA to reflect two ends of a continuum in sponge microbiome community structure. Although the four highest values of *S* and *D* were found in four of the seven HMA species in our study, two HMA species displayed very low values of *D*. Both of these sponges host photosymbionts, so these low values of *D* potentially reflect strong selection for the nutritional benefits received from these partners (Thacker and Freeman, 2012). Similar host selection for symbiont-derived benefits might also occur for other microbial OTUs in LMA sponges that display lower values of *D*. We observed strong phylogenetic signal for *D*, but BCD and phylogenetic dissimilarity were influenced more by host species identity than host phylogenetic relatedness. In contrast to previous studies, we found a low number of species-specific microbial OTUs, as well as an unexpectedly large range of intraspecific variation in BCD. In future research on these microbiomes, these metrics of community structure can be used in combination with microbial abundance to assess trends in the evolution of microbiomes. Based on our current dataset, broad-scale microbial diversity within a host sponge appears to be strongly influenced by host phylogeny, but the specific members of each host's microbial community appear to be structured by unique interactions within each host species.

### Conflict of interest statement

The authors declare that the research was conducted in the absence of any commercial or financial relationships that could be construed as a potential conflict of interest.

## Supplementary material

Supplementary Figure 1 **Mean percentage contribution of the 103 OTUs contributing at least 40% of the SIMPER contrast of Bray–Curtis dissimilarity among host species**. These data are square-root transformed for ease of visualization. OTUs are grouped by phylum across the top of the figure, with the exception of Proteobacteria, which is split into classes. The host sponge phylogeny is displayed to the left of the heat map for ease of reference.

Supplementary Figure 2 ***F*-ratio of OTU richness across host species plotted against an array of minimum read thresholds**. The *F*-ratio was significantly influenced by minimum read threshold (polynomial regression: *df* = 2, *F* = 24.03, *P* = 0.006).
