# Supplementary material for: Phylogenetic signal in the community structure of host-specific microbiomes of tropical marine sponges
Source: Front Microbiol. 2014 Oct 17;5:532. doi: 10.3389/fmicb.2014.00532 (PMC4201110; doi:10.3389/fmicb.2014.00532)
Supplement: Supplementary file 1 [file Table1.DOCX]

**Supplementary Table 1. Metadata associated with each sponge specimen analyzed in this study, including host species name, geographic location, latitude, longitude, sampling depth, host taxonomic order, EMP ID, and number of reads per specimen.**

| PortoL ID | HOST SPECIES | GEOGRAPHIC LOCATION | LONGITUDE | LATITUDE | SAMPLING DEPTH | HOST ORDER | EMP ID | NUMBER OF READS |
| --- | --- | --- | --- | --- | --- | --- | --- | --- |
| P12x105 | *Aiolochroia crassa* | Punta Caracol | -82.278 | 9.36068 | 5 | Verongida | P12x105 | 9854 |
| P12x109 | *Aiolochroia crassa* | Punta Caracol | -82.278 | 9.36068 | 5 | Verongida | P12x109 | 16121 |
| P12x110 | *Aiolochroia crassa* | Punta Caracol | -82.278 | 9.36068 | 5 | Verongida | P12x110 | 16113 |
| P12x117 | *Aiolochroia crassa* | Punta Caracol | -82.278 | 9.36068 | 5 | Verongida | P12x117 | 16140 |
| P12x120 | *Aiolochroia crassa* | Punta Caracol | -82.278 | 9.36068 | 5 | Verongida | P12x120 | 20539 |
| P12x145 | *Amphimedon compressa* | STRI Point | -82.2595 | 9.35167 | 2 | Haplosclerida | P12x145 | 8361 |
| P12x147 | *Amphimedon compressa* | STRI Point | -82.2595 | 9.35167 | 2 | Haplosclerida | P12x147 | 7351 |
| P12x149 | *Amphimedon compressa* | STRI Point | -82.2595 | 9.35167 | 2 | Haplosclerida | P12x149 | 8983 |
| P12x150 | *Amphimedon compressa* | STRI Point | -82.2595 | 9.35167 | 2 | Haplosclerida | P12x150 | 7539 |
| P12x151 | *Amphimedon compressa* | STRI Point | -82.2595 | 9.35167 | 2 | Haplosclerida | P12x151 | 11454 |
| P12x54 | *Amphimedon erina* | Solarte Mangrove | -82.17317 | 9.30583 | 2 | Haplosclerida | P12x54 | 22654 |
| P12x58 | *Amphimedon erina* | Solarte Mangrove | -82.17317 | 9.30583 | 2 | Haplosclerida | P12x58 | 15759 |
| P81 | *Amphimedon erina* | Solarte Mangrove | -82.17317 | 9.30583 | 1 | Haplosclerida | P81 | 17066 |
| SI06x67 | *Amphimedon erina* | Solarte Mangrove | -82.17317 | 9.30583 | 2 | Haplosclerida | SI06.67 | 30528 |
| SI06x79 | *Amphimedon erina* | Solarte Mangrove | -82.17317 | 9.30583 | 2 | Haplosclerida | SI06.79 | 15252 |
| P12x103 | *Aplysina cauliformis* | Punta Caracol | -82.278 | 9.36068 | 5 | Verongida | 703 | 11760 |
| P12x104 | *Aplysina cauliformis* | Punta Caracol | -82.278 | 9.36068 | 5 | Verongida | 704 | 11659 |
| P12x106 | *Aplysina cauliformis* | Punta Caracol | -82.278 | 9.36068 | 5 | Verongida | 705 | 19325 |
| P12x107 | *Aplysina cauliformis* | Punta Caracol | -82.278 | 9.36068 | 5 | Verongida | 706 | 18884 |
| P12x108 | *Aplysina cauliformis* | Punta Caracol | -82.278 | 9.36068 | 5 | Verongida | 707 | 19309 |
| P12x78 | *Aplysina fulva* | Punta Caracol | -82.278 | 9.36068 | 5 | Verongida | 708 | 22994 |
| P12x111 | *Aplysina fulva* | Punta Caracol | -82.278 | 9.36068 | 5 | Verongida | 709 | 21336 |
| P12x112 | *Aplysina fulva* | Punta Caracol | -82.278 | 9.36068 | 5 | Verongida | 710 | 15223 |
| P12x113 | *Aplysina fulva* | Punta Caracol | -82.278 | 9.36068 | 5 | Verongida | 711 | 11497 |
| P12x115 | *Aplysina fulva* | Punta Caracol | -82.278 | 9.36068 | 5 | Verongida | 712 | 8604 |
| P12x56 | *Chalinula molitba* | Solarte Mangrove | -82.17317 | 9.30583 | 1 | Haplosclerida | P12x56 | 12516 |
| SI06x80 | *Chalinula molitba* | Solarte Mangrove | -82.17317 | 9.30583 | 2 | Haplosclerida | SI06.80 | 9285 |
| SI06x69 | *Chalinula molitba* | Solarte Mangrove | -82.17317 | 9.30583 | 2 | Haplosclerida | SI06x69 | 9153 |
| P12x71 | *Chondrilla caribensis* | STRI Point | -82.2595 | 9.35167 | 2 | Hadromerida | P12x71 | 20750 |
| P12x73 | *Chondrilla caribensis* | STRI Point | -82.2595 | 9.35167 | 2 | Hadromerida | P12x73 | 14472 |
| P12x74 | *Chondrilla caribensis* | STRI Point | -82.2595 | 9.35167 | 2 | Hadromerida | P12x74 | 14938 |
| P12x75 | *Chondrilla caribensis* | STRI Point | -82.2595 | 9.35167 | 2 | Hadromerida | P12x75 | 17879 |
| P12x80 | *Chondrilla caribensis* | STRI Point | -82.2595 | 9.35167 | 2 | Hadromerida | P12x80 | 15618 |
| P31 | *Dysidea etheria* | Solarte Mangrove | -82.17317 | 9.30583 | 0 | Dictyoceratida | P31 | 6455 |
| SI06x66 | *Dysidea etheria* | Solarte Mangrove | -82.17317 | 9.30583 | 2 | Dictyoceratida | SI06.66 | 8251 |
| SI06x81 | *Dysidea etheria* | Solarte Mangrove | -82.17317 | 9.30583 | 2 | Dictyoceratida | SI06.81 | 13531 |
| SI06x93 | *Dysidea etheria* | Solarte Mangrove | -82.17317 | 9.30583 | 2 | Dictyoceratida | SI06.93 | 10626 |
| SI06x96 | *Dysidea etheria* | Solarte Mangrove | -82.17317 | 9.30583 | 2 | Dictyoceratida | SI06.96 | 9088 |
| P12x12 | *Ectyoplasia ferox* | Adrianas Reef | -82.17367 | 9.24133 | 5 | Poecilosclerida | P12x12 | 19673 |
| P12x13 | *Ectyoplasia ferox* | Adrianas Reef | -82.17367 | 9.24133 | 5 | Poecilosclerida | P12x13 | 19394 |
| P12x18 | *Ectyoplasia ferox* | Adrianas Reef | -82.17367 | 9.24133 | 5 | Poecilosclerida | P12x18 | 15709 |
| P12x19 | *Ectyoplasia ferox* | Adrianas Reef | -82.17367 | 9.24133 | 5 | Poecilosclerida | P12x19 | 12882 |
| P12x20 | *Ectyoplasia ferox* | Adrianas Reef | -82.17367 | 9.24133 | 5 | Poecilosclerida | P12x20 | 24422 |
| P12x11 | *Erylus formosus* | Adrianas Reef | -82.17367 | 9.24133 | 5 | Astrophorida | P12x11 | 20594 |
| P12x21 | *Erylus formosus* | Adrianas Reef | -82.17367 | 9.24133 | 5 | Astrophorida | P12x21 | 21769 |
| P12x22 | *Erylus formosus* | Adrianas Reef | -82.17367 | 9.24133 | 5 | Astrophorida | P12x22 | 10855 |
| P12x23 | *Erylus formosus* | Adrianas Reef | -82.17367 | 9.24133 | 5 | Astrophorida | P12x23 | 32554 |
| P12x25 | *Erylus formosus* | Adrianas Reef | -82.17367 | 9.24133 | 5 | Astrophorida | P12x25 | 12087 |
| P110 | *Haliclona tubifera* | Solarte Mangrove | -82.17317 | 9.30583 | 1 | Haplosclerida | P110 | 13046 |
| P12x51 | *Haliclona tubifera* | Solarte Mangrove | -82.17317 | 9.30583 | 1 | Haplosclerida | P12x51 | 7758 |
| SI06x94 | *Haliclona tubifera* | Solarte Mangrove | -82.17317 | 9.30583 | 2 | Haplosclerida | SI06.94 | 13141 |
| SI06x97 | *Haliclona tubifera* | Solarte Mangrove | -82.17317 | 9.30583 | 2 | Haplosclerida | SI06.97 | 28407 |
| P10x38 | *Haliclona vansoesti* | Punta Caracol | -82.278 | 9.36068 | 5 | Haplosclerida | P10X38 | 6455 |
| SI06x152 | *Haliclona vansoesti* | Adrianas Reef | -82.17367 | 9.24133 | 7 | Haplosclerida | SI06.152 | 5278 |
| P12x123 | *Iotrochota birotulata* | STRI Point | -82.261 | 9.35317 | 5 | Poecilosclerida | P12x123 | 16489 |
| P12x127 | *Iotrochota birotulata* | STRI Point | -82.261 | 9.35317 | 5 | Poecilosclerida | P12x127 | 24404 |
| P12x129 | *Iotrochota birotulata* | STRI Point | -82.261 | 9.35317 | 5 | Poecilosclerida | P12x129 | 26545 |
| P12x132 | *Iotrochota birotulata* | STRI Point | -82.261 | 9.35317 | 5 | Poecilosclerida | P12x132 | 14706 |
| P06 | *Lissodendoryx colombiensis* | STRI Point | -82.2595 | 9.35167 | 0 | Poecilosclerida | P06 | 16140 |
| P12x59 | *Lissodendoryx colombiensis* | Solarte Mangrove | -82.174 | 9.30667 | 3 | Poecilosclerida | P12x59 | 51524 |
| SI06x133 | *Lissodendoryx colombiensis* | Punta Caracol | -82.278 | 9.36068 | 10 | Poecilosclerida | SI06.133 | 18811 |
| SI06x53 | *Lissodendoryx colombiensis* | STRI Point | -82.2595 | 9.35167 | 3 | Poecilosclerida | SI06.53 | 25367 |
| SI06x54 | *Lissodendoryx colombiensis* | STRI Point | -82.2595 | 9.35167 | 3 | Poecilosclerida | SI06.54 | 49883 |
| P12x124 | *Mycale laevis* | STRI Point | -82.261 | 9.35317 | 5 | Poecilosclerida | P12x124 | 10280 |
| P12x125 | *Mycale laevis* | STRI Point | -82.261 | 9.35317 | 5 | Poecilosclerida | P12x125 | 17650 |
| P12x126 | *Mycale laevis* | STRI Point | -82.261 | 9.35317 | 5 | Poecilosclerida | P12x126 | 3908 |
| P12x130 | *Mycale laevis* | STRI Point | -82.261 | 9.35317 | 5 | Poecilosclerida | P12x130 | 13804 |
| P12x133 | *Mycale laevis* | STRI Point | -82.261 | 9.35317 | 5 | Poecilosclerida | P12x133 | 8621 |
| P12x121 | *Mycale laxissima* | STRI Point | -82.261 | 9.35317 | 5 | Poecilosclerida | P12x121 | 9202 |
| P12x122 | *Mycale laxissima* | STRI Point | -82.261 | 9.35317 | 5 | Poecilosclerida | P12x122 | 23094 |
| P12x134 | *Mycale laxissima* | STRI Point | -82.261 | 9.35317 | 5 | Poecilosclerida | P12x134 | 22264 |
| P12x143 | *Mycale laxissima* | STRI Point | -82.261 | 9.35317 | 5 | Poecilosclerida | P12x143 | 12196 |
| P12x144 | *Mycale laxissima* | STRI Point | -82.261 | 9.35317 | 5 | Poecilosclerida | P12x144 | 16122 |
| P12x101 | *Niphates erecta* | Punta Caracol | -82.278 | 9.36068 | 5 | Haplosclerida | P12x101 | 4055 |
| P12x102 | *Niphates erecta* | Punta Caracol | -82.278 | 9.36068 | 5 | Haplosclerida | P12x102 | 4989 |
| P12x116 | *Niphates erecta* | Punta Caracol | -82.278 | 9.36068 | 5 | Haplosclerida | P12x116 | 7371 |
| P12x118 | *Niphates erecta* | Punta Caracol | -82.278 | 9.36068 | 5 | Haplosclerida | P12x118 | 5064 |
| P12x119 | *Niphates erecta* | Punta Caracol | -82.278 | 9.36068 | 5 | Haplosclerida | P12x119 | 6073 |
| P20 | *Placospongia intermedia* | Adrianas Reef | -82.17367 | 9.24133 | 10 | Hadromerida | P20 | 3322 |
| SI06x73 | *Placospongia intermedia* | Solarte Mangrove | -82.17417 | 9.30683 | 2 | Hadromerida | 700 | 11051 |
| SI06x102 | *Placospongia intermedia* | Solarte Mangrove | -82.17417 | 9.30683 | 2 | Hadromerida | 701 | 17151 |
| SI06x108 | *Placospongia intermedia* | Punta Caracol | -82.278 | 9.36068 | 10 | Hadromerida | 702 | 3235 |
| BZ09x1 | *Tedania ignis* | Solarte Mangrove | -88.1045 | 16.82983 | 1 | Poecilosclerida | BZ09x1 | 31900 |
| P57 | *Tedania ignis* | Solarte Mangrove | -82.27793 | 9.38195 | 1 | Poecilosclerida | P57 | 18102 |
| SI06x27 | *Tedania ignis* | STRI Point | -82.25 | 9.33333 | 3 | Poecilosclerida | SI06.27 | 16643 |
| SI06x59 | *Tedania ignis* | STRI Point | -82.25 | 9.33333 | 3 | Poecilosclerida | SI06.59 | 15144 |
| SI06x78 | *Tedania ignis* | Solarte Mangrove | -82.17317 | 9.30583 | 2 | Poecilosclerida | SI06.78 | 15411 |
| P10x53 | *Xestospongia bocatorensis* | STRI Point | -82.2595 | 9.35167 | 4 | Haplosclerida | P10X53 | 14951 |
| SI06x11 | *Xestospongia bocatorensis* | Punta Caracol | -82.278 | 9.36068 | 10 | Haplosclerida | SI06.11 | 14475 |
| SI06x9 | *Xestospongia bocatorensis* | Punta Caracol | -82.278 | 9.36068 | 10 | Haplosclerida | SI06.9 | 21712 |
