# Supplementary material for: Phylogenetic signal in the community structure of host-specific microbiomes of tropical marine sponges
Source: Front Microbiol. 2014 Oct 17;5:532. doi: 10.3389/fmicb.2014.00532 (PMC4201110; doi:10.3389/fmicb.2014.00532)
Supplement: Supplementary file 2 [file Table2.DOCX]

**Supplementary Table 2. GenBank accession numbers of nuclear ribosomal RNA small subunit (18S) gene sequences used to construct a phylogeny of sponge hosts.**

| **Host Species** | **GenBank Accession Number** |
| --- | --- |
| *Aiolochroia crassa* | KC901954 |
| *Amphimedon compressa* | KC902400 |
| *Amphimedon erina* | JX945627 |
| *Aplysina cauliformis* | KC901896 |
| *Aplysina fulva* | KC902200 |
| *Chalinula molitba* | KC902402 |
| *Chondrilla caribensis* | KC901951 |
| *Dysidea etheria* | KC902271 |
| *Ectyoplasia ferox* | KC901974 |
| *Erylus formosus* | KC902118 |
| *Haliclona tubifera* | KC901998 |
| *Haliclona vansoesti* | KC902323 |
| *Iotrochota birotulata* | KC902147 |
| *Lissodendoryx colombiensis* | KC902105 |
| *Mycale laevis* | HQ709350 |
| *Mycale laxissima* | KC902345 |
| *Niphates erecta* | KC902280 |
| *Placospongia intermedia* | KC902020 |
| *Tedania ignis* | KC902155 |
| *Xestospongia bocatorensis* | KC902039 |
|  |  |
