# Supplementary material for: Phylogenetic signal in the community structure of host-specific microbiomes of tropical marine sponges
Source: Front Microbiol. 2014 Oct 17;5:532. doi: 10.3389/fmicb.2014.00532 (PMC4201110; doi:10.3389/fmicb.2014.00532)
Supplement: Supplementary file 3 [file Table3.DOCX]

**Supplementary Table 3.** **Mean ± standard error of univariate measures of microbiome diversity for each host species, analyzed with a minimum threshold of 1 read and rarefied to 6000 reads per sample.** *S*: OTU richness, *H’*: Shannon index, *D*: inverse Simpson index, and within-host BCD: intraspecific percentage Bray-Curtis dissimilarity.

| **Species** | ***S*** | ***H'*** | ***D*** | **within-host BCD** |
| --- | --- | --- | --- | --- |
| *Aiolochroia crassa* | 436.1 ± 68.94 | 8.23 ± 3.19 | 3.19 ± 0.54 | 33.7 ± 7.9 |
| *Amphimedon compressa* | 427.2 ± 72.47 | 11.96 ± 8.47 | 2.7 ± 0.64 | 16.2 ± 5.1 |
| *Amphimedon erina* | 591.5 ± 55.38 | 11.19 ± 5 | 3.47 ± 0.38 | 43 ± 19.2 |
| *Aplysina cauliformis* | 787.11 ± 81.92 | 65.08 ± 22.25 | 4.84 ± 0.35 | 26 ± 6.1 |
| *Aplysina fulva* | 742.6 ± 56.61 | 32.92 ± 12.26 | 4.53 ± 0.25 | 27.7 ± 6.6 |
| *Chalinula molitba* | 518.14 ± 119.68 | 17.56 ± 15.81 | 2.84 ± 1 | 24.1 ± 10.8 |
| *Chondrilla caribensis* | 680.39 ± 35.92 | 6.11 ± 1.29 | 3.59 ± 0.21 | 29.2 ± 9.1 |
| *Dysidea etheria* | 885.15 ± 93.3 | 69.19 ± 25.74 | 5.04 ± 0.3 | 66.4 ± 15.8 |
| *Ectyoplasia ferox* | 742.56 ± 114.83 | 40.91 ± 19.6 | 4.36 ± 0.74 | 24.6 ± 6.2 |
| *Erylus formosus* | 603.95 ± 97.15 | 27.89 ± 16 | 3.61 ± 0.69 | 29.7 ± 7 |
| *Haliclona tubifera* | 557.47 ± 98.43 | 16.67 ± 7.66 | 3.63 ± 0.49 | 52.5 ± 18.3 |
| *Haliclona vansoesti* | 542.59 ± 20.64 | 10.71 ± 3.67 | 3.69 ± 0.21 | 23.9 ± 19.5 |
| *Iotrochota birotulata* | 528.08 ± 213.53 | 21.4 ± 14.3 | 3.28 ± 1.01 | 4 ± 1.5 |
| *Lissodendoryx colombiensis* | 527.98 ± 41.73 | 22.57 ± 12.51 | 3.49 ± 0.58 | 42.4 ± 18.5 |
| *Mycale laevis* | 421.46 ± 74.72 | 3.22 ± 0.65 | 2.38 ± 0.26 | 48.8 ± 15.6 |
| *Mycale laxissima* | 616.74 ± 120.88 | 21.95 ± 10.43 | 3.68 ± 0.73 | 51.8 ± 13.2 |
| *Niphates erecta* | 755.34 ± 52.22 | 25.82 ± 3.49 | 4.78 ± 0.09 | 41.4 ± 10.4 |
| *Placospongia intermedia* | 951.88 ± 77 | 79.8 ± 4.19 | 5.31 ± 0.16 | 73 ± 25 |
| *Tedania ignis* | 976.5 ± 24.89 | 89.36 ± 9.94 | 5.36 ± 0.06 | 86 ± 12 |
| *Xestospongia bocatorensis* | 948.35 ± 13.87 | 81.65 ± 8.81 | 5.25 ± 0.04 | 35.9 ± 17.8 |
