# Supplementary material for: Phylogenetic signal in the community structure of host-specific microbiomes of tropical marine sponges
Source: Front Microbiol. 2014 Oct 17;5:532. doi: 10.3389/fmicb.2014.00532 (PMC4201110; doi:10.3389/fmicb.2014.00532)
Supplement: Supplementary file 4 [file Table4.DOCX]

**Supplementary Table 4. Mean ± standard error of univariate measures of microbiome diversity for each host species, analyzed with a minimum threshold of 5 reads.** *S*: OTU richness, *H’*: Shannon index, *D*: inverse Simpson index, and within-host BCD: intraspecific percentage Bray-Curtis dissimilarity.

| **Species** | ***S*** | ***H'*** | ***D*** | **within-host BCD** |
| --- | --- | --- | --- | --- |
| *Aiolochroia crassa* | 3697.8 ± 149.01 | 5.29 ± 0.08 | 73.79 ± 10.46 | 33.7 ± 7.9 |
| *Amphimedon compressa* | 1178.2 ± 95.92 | 2.76 ± 0.41 | 3.17 ± 0.71 | 16.2 ± 5.1 |
| *Amphimedon erina* | 1778.6 ± 298.01 | 2.28 ± 0.63 | 4.44 ± 2.82 | 43 ± 19.2 |
| *Aplysina cauliformis* | 4271.2 ± 176.48 | 5.57 ± 0.04 | 95.9 ± 8.79 | 26 ± 6.1 |
| *Aplysina fulva* | 3719.4 ± 203.49 | 5.35 ± 0.04 | 79.07 ± 6.01 | 27.7 ± 6.6 |
| *Chalinula molitba* | 1654 ± 101.16 | 4.85 ± 0.11 | 27.03 ± 5.1 | 24.1 ± 10.8 |
| *Chondrilla caribensis* | 2117.4 ± 108.50 | 3.96 ± 0.24 | 16.43 ± 5.63 | 29.2 ± 9.1 |
| *Dysidea etheria* | 2779.8 ± 198.78 | 4.76 ± 0.4 | 32.62 ± 12.3 | 66.4 ± 15.8 |
| *Ectyoplasia ferox* | 2500 ± 70.73 | 4.58 ± 0.09 | 35.51 ± 4.17 | 24.6 ± 6.2 |
| *Erylus formosus* | 5263.2 ± 294.78 | 5.69 ± 0.11 | 106.49 ± 21.46 | 29.7 ± 7 |
| *Haliclona tubifera* | 1946.5 ± 209.21 | 4.5 ± 0.63 | 26.69 ± 11.05 | 52.5 ± 18.3 |
| *Haliclona vansoesti* | 1117.5 ± 16.50 | 4.76 ± 0.26 | 41.75 ± 23.22 | 23.9 ± 19.5 |
| *Iotrochota birotulata* | 819 ± 55.94 | 1.57 ± 0.23 | 1.59 ± 0.13 | 4 ± 1.5 |
| *Lissodendoryx colombiensis* | 1366.2 ± 135.54 | 2.54 ± 0.15 | 3.69 ± 0.39 | 42.4 ± 18.5 |
| *Mycale laevis* | 1362 ± 106.01 | 3.69 ± 0.27 | 14.6 ± 7.79 | 48.8 ± 15.6 |
| *Mycale laxissima* | 1831.2 ± 90.58 | 3.75 ± 0.23 | 8.23 ± 1.43 | 51.8 ± 13.2 |
| *Niphates erecta* | 1109.4 ± 41.35 | 4.14 ± 0.15 | 16.42 ± 3.07 | 41.4 ± 10.4 |
| *Placospongia intermedia* | 3361.5 ± 273.54 | 5 ± 0.28 | 51.29 ± 14.23 | 73 ± 25 |
| *Tedania ignis* | 810.6 ± 42.05 | 2.11 ± 0.05 | 3.15 ± 0.4 | 86 ± 12 |
| *Xestospongia bocatorensis* | 1640.67 ± 158.86 | 3.85 ± 0.83 | 16.92 ± 9.52 | 35.9 ± 17.8 |
