# Supplementary material for: Phylogenetic signal in the community structure of host-specific microbiomes of tropical marine sponges
Source: Front Microbiol. 2014 Oct 17;5:532. doi: 10.3389/fmicb.2014.00532 (PMC4201110; doi:10.3389/fmicb.2014.00532)
Supplement: Supplementary file 6 [file DataSheet2.DOCX]

**Supplementary File 2. Summary of statistical analyses using minimum thresholds of 1 and 5 reads.**

*Minimum Threshold = 1 Read*

When including all data (equal to a minimum read threshold of a single read), diversity analysis revealed that bacterial communities were highly host specific. We found significant differences among host taxa for *S* (one-way ANOVA; df = 19, F = 8.632, P < 0.0001), *H’* (one-way ANOVA; df = 19, F = 15.95, P < 0.0001), and *D* (one-way ANOVA; df = 19, F = 14.31, P < 0.0001). We observed significant phylogenetic signal in the inverse Simpson’s index (K = 0.589, P = 0.005).

Host microbial communities showed significant taxonomic dissimilarity across host identity (adonis; df = 19, F = 7.149, R^2^ = 0.66, p = 0.001). Analysis using Mantel tests found that, when tested individually, host identity (Mantel: r = 0.436, R^2^ = 0.190, P < 0.001) and host phylogeny (Mantel: r = 0.631, R^2^ = 0.398, P < 0.001) each explained a significant amount of variability in BCD. Testing the effect of host phylogeny given host identity greatly reduced the explanatory power of phylogenetic relatedness, but remained significant (Partial Mantel: r = 0.188, R^2^ = 0.035, P < 0.001). Phylogenetic dissimilarity analysis was not possible for a dataset containing all OTUs because it exceeded the integer limits within R.

**Supplementary Table 2.1:** Adonis analysis of Bray-Curtis dissimilarity among host species for all OTUs found in the 90 sponge samples in our study.

|  | df | Sum of Squares | Mean Squares | F Model | R^2^ | P-value |
| --- | --- | --- | --- | --- | --- | --- |
| **Bray-Curtis Dissimilariy** |  |  |  |  |  |  |
| Host species identity | 19 | 26.369 | 1.388 | 7.149 | 0.6599 | 0.001 |
| Residuals | 70 | 13.59 | 0.194 |  | 0.3401 |  |
|  |  |  |  |  |  |  |

*Minimum Threshold = 5 Reads*

Diversity analyses for OTUs with a minimum threshold of 5 reads exhibited high host specificity. We found significant host specificity for *S* (one-way ANOVA; df = 19, F = 11.51, P < 0.001), *H’* (one-way ANOVA; df = 19, F = 16.12, P < 0.001), and *S* (one-way ANOVA; df = 19, F = 14.31, P < 0.001). We observed significant phylogenetic signal in the inverse Simpson’s index (K = 0.634, P = 0.002).

Host microbial communities showed significant taxonomic dissimilarity across host identity (adonis df = 19, F = 8.25, R^2^ = 0.69, p = 0.001). Analysis using Mantel tests found that, when tested individually, host identity (Mantel: r = 0.432, R^2^ = 0.186, P < 0.001) and host phylogeny (Mantel: r = 0.623, R^2^ = 0.388, P < 0.001) each explained a significant amount of variability in BCD. Testing the effect of host phylogeny given host identity greatly reduced the explanatory power of phylogenetic relatedness, but remained significant (Partial Mantel: r = 0.186, R^2^ = 0.034, P < 0.001).

Phylogenetic structuring of microbial communities was also significantly affected by host identity (adonis, df = 19, F = 43.41, R^2^ = 0.92, P = 0.001). Analysis using Mantel tests revealed that when tested individually, host identity (Mantel: r = 0.3604, R^2^ = 0.130, P < 0.001) and host phylogeny (Mantel: r = 0.3601, R^2^ = 0.130, P < 0.001) each explained a significant amount of phylogenetic dissimilarity. Testing the effect of host phylogeny given host identity reduced the explanatory power of phylogenetic relatedness, but remained significant (Partial Mantel: r = 2262, R^2^ = 0.051, P < 0.001). Taxonomic dissimilarity and phylogenetic dissimilarity are not necessarily independent of one another, and these two metrics were significantly correlated (Mantel test, R = 0.62, R^2^ = 0.384, P = 0.001).

**Supplemental Table 2.2:** Adonis analysis of Bray-Curtis dissimilarity and phylogenetic dissimilarity among host species for a minimum threshold of 5 reads.

|  | df | Sum of Squares | Mean Squares | F Model | R^2^ | P-value |
| --- | --- | --- | --- | --- | --- | --- |
| **Bray-Curtis Dissimilariy** |  |  |  |  |  |  |
| Host species identity | 19 | 27.291 | 1.436 | 8.25 | 0.691 | 0.001 |
| Residuals | 70 | 12.192 | 0.174 |  | 0.309 |  |
|  |  |  |  |  |  |  |
| **Phylogenetic Dissimilarity** |  |  |  |  |  |  |
| Host species identity | 19 | 1.07 | 0.056 | 43.406 | 0.922 | 0.001 |
| Residuals | 70 | 0.0908 | 0.001 |  | 0.078 |  |
