# Supplementary figures and images for: Phylogenetic signal in the community structure of host-specific microbiomes of tropical marine sponges
Source: Front Microbiol. 2014 Oct 17;5:532. doi: 10.3389/fmicb.2014.00532 (PMC4201110; doi:10.3389/fmicb.2014.00532)

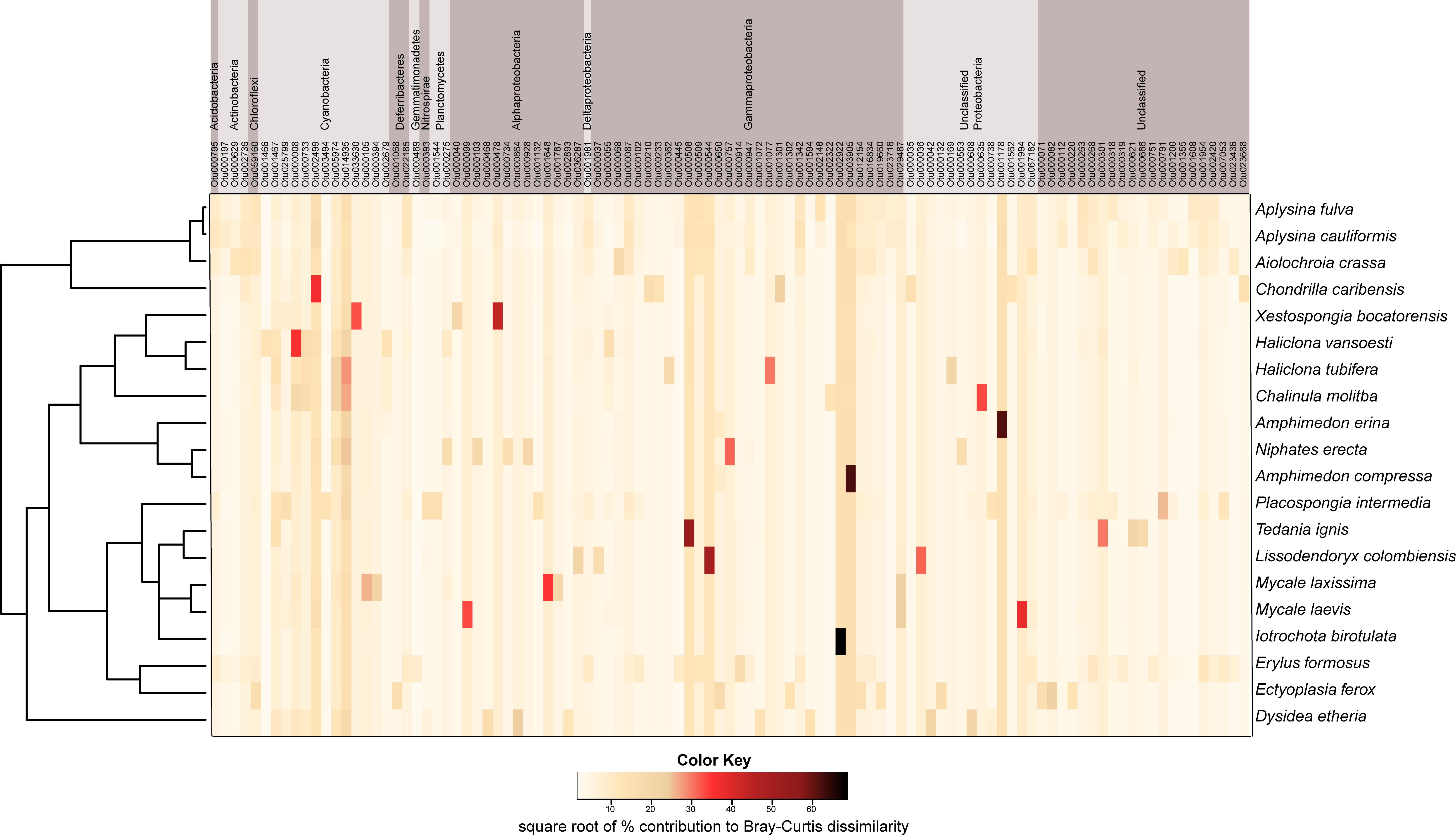

Supplement: Supplementary Figure 1 — Mean percentage contribution of the 103 OTUs contributing at least 40% of the SIMPER contrast of Bray–Curtis dissimilarity among host species. These data are square-root transformed for ease of visualization. OTUs are grouped by phylum across the top of the figure, with the exception of Proteobacteria, which is split into classes. The host sponge phylogeny is displayed to the left of the heat map for ease of reference. [file Image1.TIF]

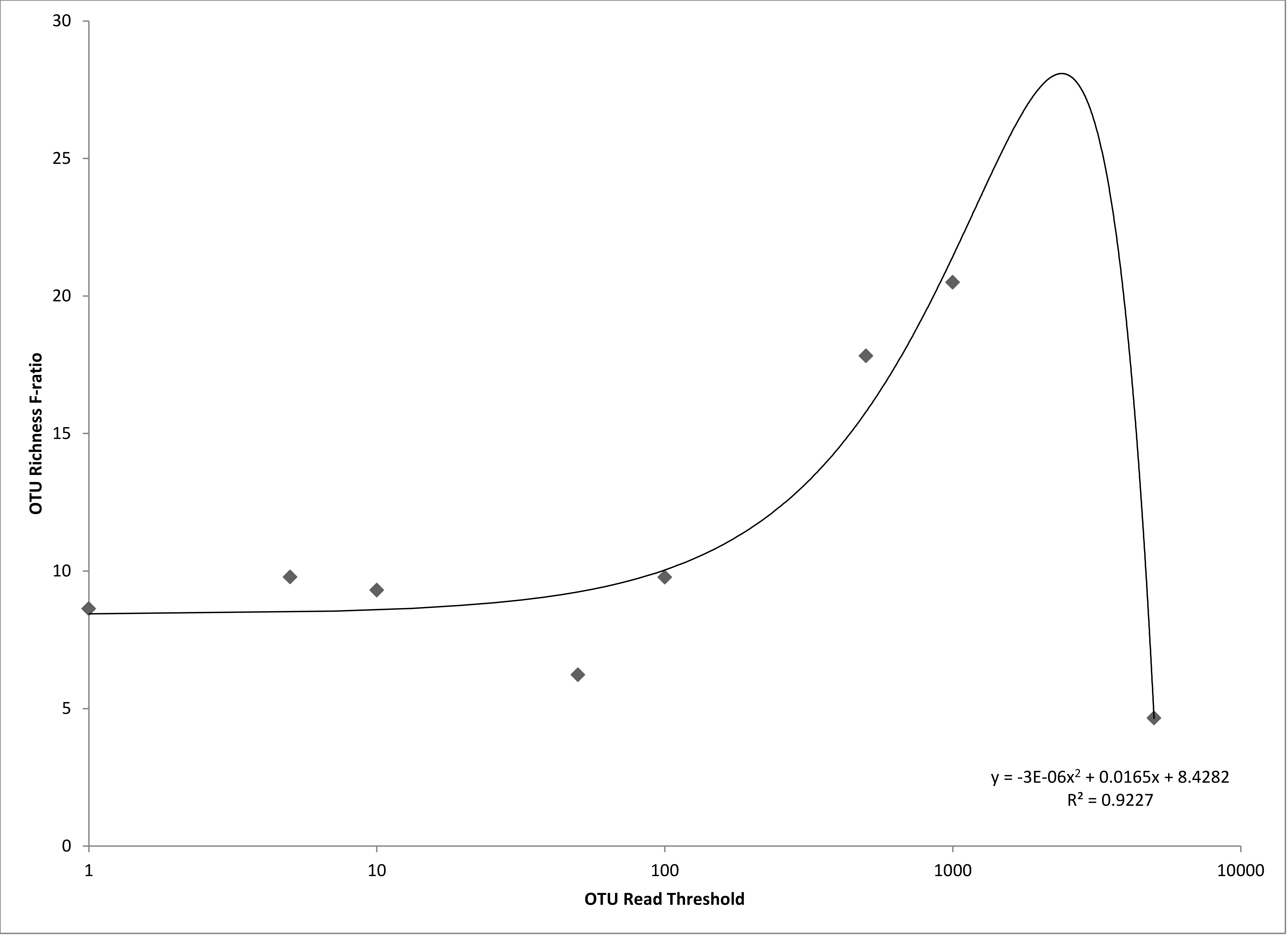

Supplement: Supplementary Figure 2 — F-ratio of OTU richness across host species plotted against an array of minimum read thresholds. The F-ratio was significantly influenced by minimum read threshold (polynomial regression: df = 2, F = 24.03, P = 0.006). [file Image2.TIF]
